# What Causes Stuttering?

**DOI:** 10.1371/journal.pbio.0020046

**Published:** 2004-02-17

**Authors:** Christian Büchel, Martin Sommer

## Abstract

The mystery of a sometimes debilitating speech disorder is examined by cognitive neuroscientists

Stuttering, with its characteristic disruption in verbal fluency, has been known for centuries; earliest descriptions probably date back to the Biblical Moses' “slowness of speech and tongue” and his related avoidance behavior (Exodus 4, 10–13). Stuttering occurs in all cultures and ethnic groups (Andrews et al. 1983; Zimmermann et al. 1983), although prevalence might differ. Insofar as many of the steps in how we produce language normally are still a mystery, disorders like stuttering are even more poorly understood. However, genetic and neurobiological approaches are now giving us clues to causes and better treatments.

## What Is Stuttering?

**Figure 1:**
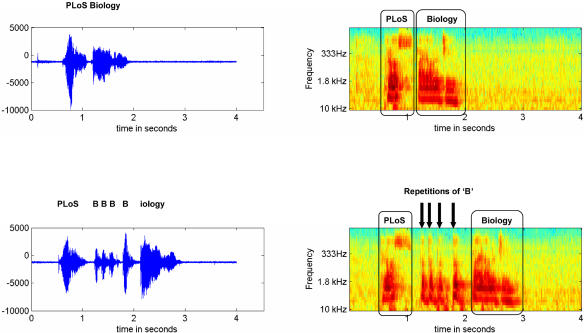
Speech Waveforms and Sound Spectrograms of a Male Speaker Saying “PLoS Biology” The left column shows speech waveforms (amplitude as a function of time); the right column shows a time–frequency plot using a wavelet decomposition of these data. In the top row, speech is fluent; in the bottom row, stuttering typical repetitions occur at the “B” in “Biology.” Four repetitions can be clearly identified (arrows) in the spectrogram (lower right).

Stuttering is a disruption in the fluency of verbal expression characterized by involuntary, audible or silent, repetitions or prolongations of sounds or syllables (Figure 1). These are not readily controllable and may be accompanied by other movements and by emotions of negative nature such as fear, embarrassment, or irritation (Wingate 1964). Strictly speaking, stuttering is a symptom, not a disease, but the term *stuttering* usually refers to both the disorder and symptom.

Developmental stuttering evolves before puberty, usually between two and five years of age, without apparent brain damage or other known cause (“idiopathic”). It is important to distinguish between this persistent developmental stuttering (PDS), which we focus on here, and acquired stuttering. Neurogenic or acquired stuttering occurs after a definable brain damage, e.g., stroke, intracerebral hemorrhage, or head trauma. It is a rare phenomenon that has been observed after lesions in a variety of brain areas (Grant et al. 1999; Ciabarra et al. 2000).

The clinical presentation of developmental stuttering differs from acquired stuttering in that it is particularly prominent at the beginning of a word or a phrase, in long or meaningful words, or syntactically complex utterances (Karniol 1995; Natke et al. 2002), and the associated anxiety and secondary symptoms are more pronounced (Ringo and Dietrich 1995). Moreover, at repeated readings, stuttering frequency tends to decline (adaptation) and to occur at the same syllables as before (consistency). Nonetheless, the distinction between both types of stuttering is not strict. In children with perinatal or other brain damage, stuttering is more frequent than in age-matched controls, and both types of stuttering may overlap (Andrews et al. 1983).

## Who Is Affected?

PDS is a very frequent disorder, with approximately 1% of the population suffering from this condition. An estimated 3 million people in the United States and 55 million people worldwide stutter. Prevalence is similar in all social classes. In many cases, stuttering severely impairs communication, with devastating socioeconomic consequences. However, there are also many stutterers who, despite their disorder, have become famous. For instance, Winston Churchill had to rehearse all his public speeches to perfection and even practiced answers to possible questions and criticisms to avoid stuttering. Charles Darwin also stuttered; interestingly, his grandfather Erasmus Darwin suffered from the same condition, highlighting the fact that stuttering runs in families and is likely to have a genetic basis.

The incidence of PDS is about 5%, and its recovery rate is up to about 80%, resulting in a prevalence of PDS in about 1% of the adult population. As recovery is considerably more frequent in girls than in boys, the male-to-female ratio increases during childhood and adolescence to reach three or four males to every one female in adulthood. It is not clear to what extent this recovery is spontaneous or induced by early speech therapy. Also, there is no good way of predicting whether an affected child will recover (Yairi and Ambrose 1999).

The presence of affected family members suggests a hereditary component. The concordance rate is about 70% for monozygotic twins (Andrews et al. 1983; Felsenfeld et al. 2000), about 30% for dizygotic twins (Andrews et al. 1983; Felsenfeld et al. 2000), and 18% for siblings of the same sex (Andrews et al. 1983). Given the high recovery rate, it may well be that the group abnormalities observed in adults reflects impaired recovery rather than the causes of stuttering (Andrews et al. 1983).

## Changing Theories

**Figure 2:**
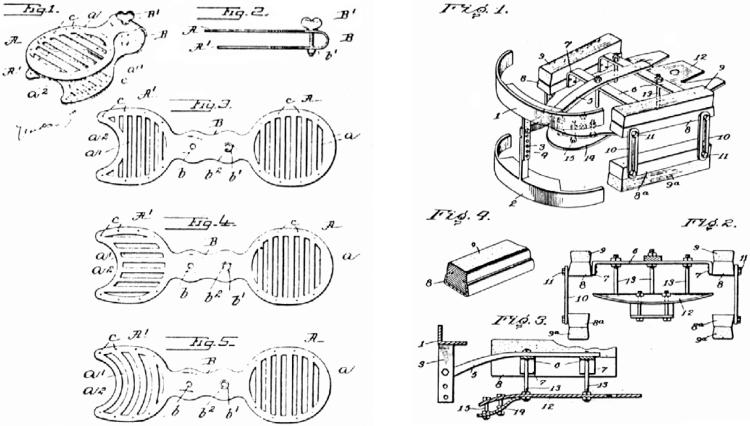
Two Different Apparatuses to Prevent Stuttering On the left is a device by Gardner from 1899 to artificially add weight to the tongue (United States patent number 625,879). On the right is a more complex speech apparatus by Peate from 1912 (United States patent number 1,030,964).

Over the centuries, a variety of theories about the origin of stuttering and corresponding treatment approaches have been proposed. In ancient Greece, theories referred to dryness of the tongue. In the 19^th^ century, abnormalities of the speech apparatus were thought to cause stuttering. Thus, treatment was based on extensive “plastic” surgery, often leading to mutilations and additional disabilities. Other treatment options were tongue-weights or mouth prostheses (Katz 1977) (Figure 2). In the 20th century, stuttering was primarily thought to be a psychogenic disorder. Consequently, psychoanalytical approaches and behavioral therapy were applied to solve possible neurotic conflicts (Plankers 1999). However, studies of personality traits and child–parent interactions did not detect psychological patterns consistently associated with stuttering (Andrews et al. 1983).

Other theories regard stuttering as a learned behavior resulting from disadvantageous external, usually parental, reactions to normal childhood dysfluencies (Johnson 1955). While this model has failed to explain the core symptoms of stuttering (Zimmermann et al. 1983), it may well explain secondary symptoms (Andrews et al. 1983), and guided early parental intervention may prevent persistence into adulthood (Onslow et al. 2001). The severity of PDS is clearly modulated by arousal, nervousness, and other factors (Andrews et al. 1983). This has led to a two-factor model of PDS. The first factor is believed to cause the disorder and is most likely a structural or functional central nervous system (CNS) abnormality, whereas the second factor reinforces the first one, especially through avoidance learning. However, one should be careful to call the latter factor “psychogenic” or “psychological,” because neuroscience has shown that learning is not simply “psychogenic” but leads to measurable changes in the brain (Kandel and O'Dell 1992).

In some cases, arousal actually improves stuttering instead of making it worse. Consequently, some famous stutterers have “treated” their stuttering by putting themselves on the spot. Anecdotally, the American actor Bruce Willis, who began stuttering at the age of eight, joined a drama club in high school and his stuttering vanished in front of an audience.

## Is Stuttering a Sensory, Motor, or Cognitive Disorder?

Stuttering subjects as a group differ from fluent control groups by showing, on average, slightly lower intelligence scores on both verbal and nonverbal tasks and by delays in speech development (Andrews et al. 1983; Paden et al. 1999). However, decreased intelligence scores need to be interpreted carefully, as stutterers show a schooling disadvantage of several months (Andrews et al. 1983). Associated symptoms comprise delays in tasks requiring a vocal response (Peters et al. 1989) and in complex bimanual timed tasks such as inserting a string in the eye of a needle (Vaughn and Webster 1989), whereas many other studies on sensory–motor reaction times yielded inconsistent results (Andrews et al. 1983). Alterations of auditory feedback (e.g., delayed auditory feedback, frequency-altered feedback), various forms of other auditory stimulation (e.g., chorus reading), and alteration of speech rhythm (e.g., syllable-timed speech) yield a prompt and marked reduction of stuttering frequency, which has raised suspicions of impaired auditory processing or rhythmic pacemaking in stuttering subjects (Lee 1951; Brady and Berson 1975; Hall and Jerger 1978; Salmelin et al. 1998). Other groups have also reported discoordinated and delayed onset of complex articulation patterns in stuttering subjects (Caruso et al. 1988; van Lieshout et al. 1993). The assumption that stuttering might be a form of dystonia—involuntary muscle contractions produced by the CNS—specific to language production (Kiziltan and Akalin 1996) was not supported by a study on motor cortex excitability (Sommer et al. 2003).

Neurochemistry, however, may link stuttering with disorders of a network of structures involved in the control of movement, the basal ganglia. An increase of the neurotransmitter dopamine has been associated with movement disorders such as Tourette syndrome (Comings et al. 1996; Abwender et al. 1998), which is a neurological disorder characterized by repeated and involuntary body movements and vocal sounds (motor and vocal tics). Accordingly, like Tourette syndrome, stuttering improves with antidopaminergic medication, e.g., neuroleptics such as haloperidol, risperidone, and olanzapine (Brady 1991; Lavid et al. 1999; Maguire et al. 2000), and anecdotal reports suggest that it is accentuated or appears under treatment with dopaminergic medication (Koller 1983; Anderson et al. 1999; Shahed and Jankovic 2001). Hence, a hyperactivity of the dopaminergic neurotransmitter system has been hypothesized to contribute to stuttering (Wu et al. 1995). Although dopamine antagonists have a positive effect on stuttering, they all have side effects that have prevented them from being a first line treatment of stuttering.

## Lessons from Imaging the Brain

Given reports on acquired stuttering after brain trauma (Grant et al. 1999; Ciabarra et al. 2000), one might think that a lesion analysis (i.e., asking the question where do all lesions that lead to stuttering overlap) could help to find the location of an abnormality linked to stuttering. Unfortunately, lesions leading to stuttering are widespread and do not seem to follow an overlapping pattern. Even the contrary has been observed, a thalamic stroke after which stuttering was “cured” in a patient (Muroi et al. 1999).

In fluent speakers, the left language-dominant brain hemisphere is most active during speech and language tasks. However, early studies on EEG lateralization already strongly suggested abnormal hemispheric dominance (Moore and Haynes 1980) in stutterers. With the advent of other noninvasive brain imaging techniques like positron emission tomography (PET) and functional magnetic resonance imaging (fMRI), it became possible to visualize brain activity of stutterers and compare these patterns to fluent controls. Following prominent theories that linked stuttering with an imbalance of hemispherical asymmetry (Travis 1978; Moore and Haynes 1980), an important PET study (Fox et al. 1996) reported increased activation in the right hemisphere in a language task in developmental stutterers. Another PET study (Braun et al. 1997) confirmed this result, but added an important detail to the previous study: Braun and colleagues found that activity in the left hemisphere was more active during the production of stuttered speech, whereas activation of the right hemisphere was more correlated with fluent speech. Thus, the authors concluded that the primary dysfunction is located in the left hemisphere and that the hyperactivation of the right hemisphere might not be the cause of stuttering, but rather a compensatory process. A similar compensatory process has been observed after stroke and aphasia, where an intact right hemisphere can at least partially compensate for a loss of function (Weiller et al. 1995). Right hemisphere hyperactivation during fluent speech has been more recently confirmed with fMRI (Neumann et al. 2003).

PET and fMRI have high spatial resolution, but because they only indirectly index brain activity through blood flow, their temporal resolution is rather limited. Magnetoencephalography (MEG) is the method of choice to investigate fine-grained temporal sequence of brain activity. Consequently, MEG was used to investigate stutterers and fluent controls reading single words (Salmelin et al. 2000). Importantly, stutterers were reported to have read most single words fluently. Nevertheless, the data showed a clear-cut difference between stutterers and controls. Whereas fluent controls activated left frontal brain areas involved in language planning before central areas involved in speech execution, this pattern was absent, even reversed, in stutterers. This was the first study to directly show a neuronal correlate of a hypothesized speech timing disorder in stutterers (Van Riper 1982).

Thus, functional neuroimaging studies have revealed two important facts: (i) in stutterers, the right hemisphere seems to be hyperactive, and (ii) a timing problem seems to exist between the left frontal and the left central cortex. The latter observation also fits various observations that have shown that stutterers have slight abnormalities in complex coordination tasks, suggesting that the underlying problem is located around motor and associated premotor brain areas.

Are there structural abnormalities that parallel the functional abnormalities? The first anatomical study to investigate this question used high-resolution MR scans and found abnormalities of speech–language areas (Broca's and Wernicke's area) (Foundas et al. 2001). In addition, these researchers reported abnormalities in the gyrification pattern. Gyrification is a complex developmental procedure, and abnormalities in this process are an indicator of a developmental disorder.

**Figure 3:**
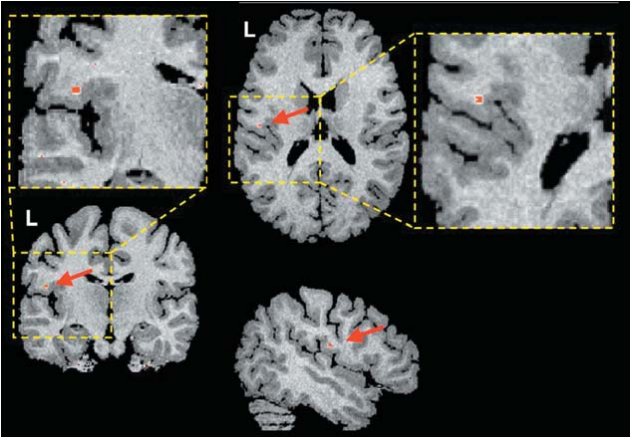
Decreased Fiber Coherence Decreased fiber coherences, as observed with DTI, in persistent developmental stutterers compared with a fluent control group. A red dot indicates the peak difference in a coronal (top left), axial (top right), and a sagittal (bottom) slice.

Another recent study investigated the hypothesis that impaired cortical connectivity might underlie timing disturbances between frontal and central brain regions observed in MEG studies (Figure 3). Using a new MRI technique, diffusion tensor imaging (DTI), that allows the assessment of white matter ultrastructure, investigators saw an area of decreased white matter tract coherence in the Rolandic operculum (Sommer et al. 2002). This structure is adjacent to the primary motor representation of tongue, larynx, and pharynx (Martin et al. 2001) and the inferior arcuate fascicle linking temporal and frontal language areas, which both form a temporofrontal language system involved in word perception and production (Price et al. 1996). It is thus conceivable that disturbed signal transmission through fibers passing the left Rolandic operculum impairs the fast sensorimotor integration necessary for fluent speech production. This theory also explains why the normal temporal pattern of activation between premotor and motor cortex is disturbed (Salmelin et al. 2000) and why, as a consequence, the right hemisphere language areas try to compensate for this deficit (Fox et al. 1996).

These new data also provide a theory to explain the mechanism of common fluency-inducing maneuvers like chorus reading, singing, and metronome reading that reduce stuttering instantaneously. All these procedures involve an external signal (i.e., other readers in chorus reading, the music in singing, and the metronome itself). All these external signals feed into the “speech production system” through the auditory cortex. It is thus possible that this external trigger signal reaches speech-producing central brain areas by circumventing the frontocentral disconnection and is able to resynchronize frontocentral decorrelated activity. In simple terms, these external cues can be seen as an external “pacemaker.”

## Future Directions in Research

There are numerous outstanding issues in stuttering. If structural changes in the brain cause PDS, the key question is when this lesion appears. Although symptoms are somewhat different, it would be interesting to find out to what extent transient stuttering (which occurs in 3%–5% in childhood) is linked to PDS. It is possible that all children who show signs of stuttering develop a structural abnormality during development, but this is transient in those who become fluent speakers. If this is the case, it is even more important that therapy starts as early as possible if it is to have most impact. This question can now be answered with current methodology, i.e., noninvasive brain imaging using MRI.

Given that boys are about four times less likely to recover from stuttering than girls, it is tempting to speculate that all stutterers have a slight abnormality, but only those that can use the right hemisphere for language can develop into fluent speakers. Language lateralization is less pronounced in women (McGlone 1980) and might therefore be related to the fact that women show an overall lower incidence in PDS. Again, a developmental study comparing children who stutter with fluent controls and, most importantly, longitudinal studies on these children should be able to answer these questions.

It is unlikely that stuttering is inherited in a simple fashion. Currently, a multifactorial model for genetic transmission is most likely. Moreover, it is unclear whether a certain genotype leads to stuttering or only represents a risk factor and that other environmental factors are necessary to develop PDS. Again, this question might be answered in the near future, as the National Institutes of Health has recently completed the data collection phase of a large stuttering sample for genetic linkage analysis.

## Abbreviations

CNS: central nervous system
DTI: diffusion tensor imaging
fMRI: functional magnetic resonance imaging
MEG: magnetoencephalography
MRI: magnetic resonance imaging
PDS: persistent developmental stuttering
PET: positron emission tomography

## References

[pbio-0020046-Abwender1] Abwender DA, Trinidad KS, Jones KR, Como PG, Hymes E (1998). Features resembling Tourette's syndrome in developmental stutterers. Brain Lang.

[pbio-0020046-Anderson1] Anderson JM, Hughes JD, Rothi LJ, Crucian GP, Heilman KM (1999). Developmental stuttering and Parkinson's disease: The effects of levodopa treatment. J Neurol Neurosurg Psychiatry.

[pbio-0020046-Andrews1] Andrews G, Craig A, Feyer AM, Hoddinott S, Howie P (1983). Stuttering: A review of research findings and theories circa 1982. J Speech Hear Disord.

[pbio-0020046-Brady1] Brady JP (1991). The pharmacology of stuttering: A critical review. Am J Psychiatry.

[pbio-0020046-Brady2] Brady JP, Berson J (1975). Stuttering, dichotic listening, and cerebral dominance. Arch Gen Psychiatry.

[pbio-0020046-Braun1] Braun AR, Varga M, Stager S, Schulz G, Selbie S (1997). Altered patterns of cerebral activity during speech and language production in developmental stuttering: An H2(15)O positron emission tomography study. Brain.

[pbio-0020046-Caruso1] Caruso AJ, Abbs JH, Gracco VL (1988). Kinematic analysis of multiple movement coordination during speech in stutterers. Brain.

[pbio-0020046-Ciabarra1] Ciabarra AM, Elkind MS, Roberts JK, Marshall RS (2000). Subcortical infarction resulting in acquired stuttering. J Neurol Neurosurg Psychiatry.

[pbio-0020046-Comings1] Comings DE, Wu S, Chiu C, Ring RH, Gade R (1996). Polygenic inheritance of Tourette syndrome, stuttering, attention deficit hyperactivity, conduct, and oppositional defiant disorder: The additive and subtractive effect of the three dopaminergic genes—DRD2, DßH, and DAT1. Am J Med Genet.

[pbio-0020046-Felsenfeld1] Felsenfeld S, Kirk KM, Zhu G, Statham DJ, Neale MC (2000). A study of the genetic and environmental etiology of stuttering in a selected twin sample. Behav Genet.

[pbio-0020046-Foundas1] Foundas AL, Bollich AM, Corey DM, Hurley M, Heilman KM (2001). Anomalous anatomy of speech–language areas in adults with persistent developmental stuttering. Neurology.

[pbio-0020046-Fox1] Fox PT, Ingham RJ, Ingham JC, Hirsch TB, Downs JH (1996). A PET study of the neural systems of stuttering. Nature.

[pbio-0020046-Grant1] Grant AC, Biousse V, Cook AA, Newman NJ (1999). Stroke-associated stuttering. Arch Neurol.

[pbio-0020046-Hall1] Hall JW, Jerger J (1978). Central auditory function in stutterers. J Speech Hear Res.

[pbio-0020046-Johnson1] Johnson W, Johnson W, Leutenegger RR (1955). A study on the onset and development of stuttering. Stuttering in children and adults: Thirty years of research at the University of Iowa.

[pbio-0020046-Kandel1] Kandel ER, O'Dell TJ (1992). Are adult learning mechanisms also used for development?. Science.

[pbio-0020046-Karniol1] Karniol R (1995). Stuttering, language, and cognition: A review and a model of stuttering as suprasegmental sentence plan alignment (SPA). Psychol Bull.

[pbio-0020046-Katz1] Katz M (1977). Survey of patented anti-stuttering devices. J Commun Disord.

[pbio-0020046-Kiziltan1] Kiziltan G, Akalin MA (1996). Stuttering may be a type of action dystonia. Mov Disord.

[pbio-0020046-Koller1] Koller WC (1983). Dysfluency (stuttering) in extrapyramidal disease. Arch Neurol.

[pbio-0020046-Lavid1] Lavid N, Franklin DL, Maguire GA (1999). Management of child and adolescent stuttering with olanzapine: Three case reports. Ann Clin Psychiatry.

[pbio-0020046-Lee1] Lee BS (1951). Artificial stutter. J Spech Hear Dis.

[pbio-0020046-Maguire1] Maguire GA, Riley GD, Franklin DL, Gottschalk LA (2000). Risperidone for the treatment of stuttering. J Clin Psychopharmacol.

[pbio-0020046-Martin1] Martin RE, Goodyear BG, Gati JS, Menon RS (2001). Cerebral cortical representation of automatic and volitional swallowing in humans. J Neurophysiol.

[pbio-0020046-McGlone1] McGlone J (1980). Sex differences in human brain asymmetry: A critical survey. Behav Brain Sci.

[pbio-0020046-Moore1] Moore WH, Haynes WO (1980). Alpha hemispheric asymmetry and stuttering: Some support for a segmentation dysfunction hypothesis. J Speech Hear Res.

[pbio-0020046-Muroi1] Muroi A, Hirayama K, Tanno Y, Shimizu S, Watanabe T (1999). Cessation of stuttering after bilateral thalamic infarction. Neurology.

[pbio-0020046-Natke1] Natke U, Grosser J, Sandrieser P, Kalveram KT (2002). The duration component of the stress effect in stuttering. J Fluency Disord.

[pbio-0020046-Neumann1] Neumann K, Euler HA, Gudenberg AW, Giraud AL, Lanfermann H (2003). The nature and treatment of stuttering as revealed by fMRI: A within- and between-group comparison. J Fluency Disord.

[pbio-0020046-Onslow1] Onslow M, Menzies RG, Packman A (2001). An operant intervention for early stuttering: The development of the Lidcombe program. Behav Modif.

[pbio-0020046-Paden1] Paden EP, Yairi E, Ambrose NG (1999). Early childhood stuttering. II. Initial status of phonological abilities. J Speech Lang Hear Res.

[pbio-0020046-Peters1] Peters HF, Hulstijn W, Starkweather CW (1989). Acoustic and physiological reaction times of stutterers and nonstutterers. J Speech Hear Res.

[pbio-0020046-Plankers1] Plankers T (1999). Speaking in the claustrum: The psychodynamics of stuttering. Int J Psychoanal.

[pbio-0020046-Price1] Price CJ, Wise RJ, Warburton EA, Moore CJ, Howard D (1996). Hearing and saying: The functional neuro-anatomy of auditory word processing. Brain.

[pbio-0020046-Ringo1] Ringo CC, Dietrich S (1995). Neurogenic stuttering: An analysis and critique. J Med Speech Lang Path.

[pbio-0020046-Salmelin1] Salmelin R, Schnitzler A, Schmitz F, Jäncke L, Witte OW (1998). Functional organization of the auditory cortex is different in stutterers and fluent speakers. Neuroreport.

[pbio-0020046-Salmelin2] Salmelin R, Schnitzler A, Schmitz F, Freund HJ (2000). Single word reading in developmental stutterers and fluent speakers. Brain.

[pbio-0020046-Shahed1] Shahed J, Jankovic J (2001). Re-emergence of childhood stuttering in Parkinson's disease: A hypothesis. Mov Disord.

[pbio-0020046-Sommer1] Sommer M, Koch MA, Paulus W, Weiller C, Buechel C (2002). A disconnection of speech-relevant brain areas in developmental stuttering. Lancet.

[pbio-0020046-Sommer2] Sommer M, Wischer S, Tergau F, Paulus W (2003). Normal intracortical excitability in developmental stuttering. Mov Disord.

[pbio-0020046-Travis1] Travis LE (1978). The cerebral dominance theory of stuttering, 1931–1978. J Speech Hear Disord.

[pbio-0020046-vanLieshout1] van Lieshout PH, Peters HF, Starkweather CW, Hulstijn W (1993). Physiological differences between stutterers and nonstutterers in perceptually fluent speech: EMG amplitude and duration. J Speech Hear Res.

[pbio-0020046-VanRiper1] Van Riper C (1982). The nature of stuttering.

[pbio-0020046-Vaughn1] Vaughn CL, Webster WG (1989). Bimanual handedness in adults who stutter. Percept Mot Skills.

[pbio-0020046-Weiller1] Weiller C, Isensee C, Rijntjes M, Huber W, Muller S (1995). Recovery from Wernicke's aphasia: A positron emission tomographic study. Ann Neurol.

[pbio-0020046-Wingate1] Wingate ME (1964). A standard definition of stuttering. J Speech Hear Dis.

[pbio-0020046-Wu1] Wu JC, Maguire G, Riley G, Fallon J, LaCasse L (1995). A positron emission tomography [18F]deoxyglucose study of developmental stuttering. Neuroreport.

[pbio-0020046-Yairi1] Yairi E, Ambrose NG (1999). Early childhood stuttering. I. Persistency and recovery rates. J Speech Lang Hear Res.

[pbio-0020046-Zimmermann1] Zimmermann G, Liljeblad S, Frank A, Cleeland C (1983). The Indians have many terms for it: Stuttering among the Bannock–Shoshoni. J Speech Hear Res.

